# Geographical Aspects of Recent Trends in Drug-Related Deaths, with a Focus on Intra-National Contextual Variation

**DOI:** 10.3390/ijerph17218081

**Published:** 2020-11-02

**Authors:** Peter Congdon

**Affiliations:** School of Geography, Queen Mary University of London, Mile End Rd, Bethnal Green, London E1 4NS, UK; p.congdon@qmul.ac.uk

**Keywords:** mortality, contextual, opioid, drug dependence, small area

## Abstract

Background. Recent worldwide estimates are of 53 million users of opioids annually, and of 585,000 drug-related deaths, of which two thirds are due to opioids. There are considerable international differences in levels of drug death rates and substance abuse. However, there are also considerable variations within countries in drug misuse, overdose rates, and in drug death rates particularly. Wide intra-national variations characterize countries where drug deaths have risen fastest in recent years, such as the US and UK. Drug deaths are an outcome of drug misuse, which can ideally be studied at a relatively low spatial scale (e.g., US counties). The research literature suggests that small area variations in drug deaths to a considerable degree reflect contextual (place-related) factors as well as individual risk factors. Methods. We consider the role of area social status, social cohesion, segregation, urbanicity, and drug supply in an ecological regression analysis of county differences in drug deaths in the US during 2015–2017. Results. The analysis of US small area data highlights a range of factors which are statistically significant in explaining differences in drug deaths, but with no risk factor having a predominant role. Comparisons with other countries where small area drug mortality data have been analyzed show differences between countries in the impact of different contextual factors, but some common themes. Conclusions. Intra-national differences in drug-related deaths are considerable, but there are significant research gaps in the evidence base for small area analysis of such deaths.

## 1. Introduction

The United Nations Office on Drugs and Crime (UNODC) estimates that there are 53 million users of opioids annually and that 585,000 people die annually worldwide (as a contributory or direct cause) due to drug misuse [1]. Two thirds of these deaths are related to opioids. Drug deaths are a major component of the disease burden associated with drug misuse [2] and, due to their disproportionate contribution to premature deaths, have been linked to the deceleration or reversal of improvement in life expectancies [3]. 

There are considerable international differences in drug misuse prevalence, drug use patterns, and drug-related death rates. For example, the recent upturn in drug deaths in the US and Canada has been linked to growing use of fentanyl, a synthetic opioid with potentially lethal effects at low doses [4]. By contrast, in parts of Asia and Africa, growth of drug dependence has been linked to tramadol, a less lethal opioid ([5], pp. 23–26). 

Variations in drug-related deaths within countries are also pronounced and generally less well documented. Research into reasons for such variations is also relatively limited. Where data are available, they show wide disparities. For example, drug overdose mortality rates in US states (2017 data) vary 7-fold, from 8.1 per 100 thousand in Nebraska to 57.8 per 100 thousand in West Virginia [6]. In Scotland, drug death rates (for 2014–2018) by local authority range from 5 per 100,000 (Orkneys) to 31 per 100,000 (Dundee) [7]. 

As discussed in the literature on risk environments [8,9], such wide contrasts are difficult to explain solely in terms of the individual risk factor pattern of different area populations (also known as compositional factors), and contextual factors (place effects, due, for example, to structural economic shifts) are relevant also [10,11]. 

In some ways, the contextual–compositional distinction may be reductionist, and recognition of broader influences on individual behaviours is needed [12,13]. For example, under a relational perspective, context and composition are not mutually exclusive since the health status of places results from interactions of people with the wider environment. Bambra et al. [13] argue for a political economy approach which recognizes the role of structural drivers of geographic health inequalities. For example, in the case of drug-related mortality in the US, these might include variations between US states in drug law enforcement, drug addiction treatment, and naloxone accessibility [14].

Below, we discuss in more detail the main themes in the research literature on small area variations in drug-related deaths and summarize the findings of the ecological (area-based) studies on the factors associated with such variation. As an illustrative example of the issues involved, we then consider variation in drug deaths between 3141 US counties during the period 2015–2017. We use a Bayesian regression method [15], and find the relative risk of drug deaths to be especially associated with area income contrasts and social capital, with lesser impacts of ethnic segregation, unemployment, urbanicity, and drug supply. We also find evidence of considerable spatial clustering in drug deaths.

## 2. Factors Underlying Intra-National Variation in Drug-Related Deaths 

Interlinked themes are present in the literature on contextual variations in drug-related deaths within countries. Among recurring themes is the notion of “deaths of despair”, combining drug and alcohol-related deaths and suicide. This concept has been applied particularly to the United States [16,17] but has relevance for other high-income countries with high increases in drug deaths, including parts of the UK [18]. 

A predominant theme in the literature relating to deaths of despair is the effects of job losses (especially male jobs) due to de-industrialization and associated reductions in community cohesion and economic opportunity, for example, in US rust belt areas. Hence, it may be expected that unemployment contrasts between small areas, or changes in unemployment, would be associated with drug-related deaths [19]. The impact of economic restructuring is arguably especially on white males, and may partly explain the male excess in drug deaths as well as adverse trends in mortality [20]. Thus, as summarized in [21], “less-educated white males suffer overdose deaths at such a high rate that it has lowered their overall life expectancy”. 

However, there have been skeptical studies regarding the central role of de-industrialization [22], and other aspects of employment change, such as job losses related to international trade, have been proposed as a source of geographic variation in US drug deaths [21].

Alternative, more broadly based socio-economic factors have been proposed, such as material deprivation and income segregation. Thus, Boardman et al. [23] find neighborhood poverty acting to raise drug misuse, even after individual risk factors are controlled for, while Monnat [24] considers a range of indices of distress and forms of labor market dependence as potential sources of varying drug death rates in US counties over 2006–2015. 

As to UK evidence, a study of drug deaths in England and Wales [25] found a “steep socio-economic gradient” in drug deaths, while an official review [26] of drug deaths in Scotland argues that “the single biggest structural driver of problem drug use is poverty and deprivation”, later amplifying that “it is not necessarily the case that poverty in itself is a direct driver of problematic drug use; however, those in poverty are more likely to be exposed to additional risk factors, such as unstable home life, unemployment, and adverse childhood experiences which increase the likelihood of a person being predisposed towards problematic substance use”. 

The social environment, as measured by social capital and social cohesion, may affect associations between neighborhood-level poverty and drug misuse rates [27,28]. In particular, social cohesion may act as a protective factor that moderates the impact of poverty [29], with lower misuse and mortality in deprived areas that are relatively cohesive, but higher misuse and mortality where deprivation is reinforced by low cohesion. Thus, Aslund and Nillson [30] report in a Swedish study of adolescent substance use that “subjects within the group with low neighborhood social capital had … more than double the odds of having used illicit drugs compared with individuals with high neighborhood social capital”. Regarding US states, we find a correlation of −0.34 between age adjusted drug-related death rates over 2015–2017 and state level averages of social capital, extracted from https://aese.psu.edu/nercrd/community/social-capital-resources [31]. However, not all studies report a significant impact of social capital on drug misuse and overdose, with Gatti et al. [32] finding that drug overdose rates in Italian provinces were mostly determined by area socio-economic status.

Poverty effects may also be amplified by racial segregation, as mostly US studies (e.g., [33,34]) show. Segregation may impact on mental health, with Cooper et al. [33] mentioning that “black residents of segregated communities are at elevated risk of depression, anxiety, and general psychological distress”. Effects of segregation may extend beyond ethnic segregation to include measures of social segregation more generally, such as poverty segregation [34] and income inequality [35].

The urban status of small areas may also be relevant to drug-related deaths. The broader literature on drug deaths suggests that drug-related mortality may be lower in rural areas, especially after area socio-economic status is allowed for (e.g., in the USA, this would be after allowing for the impact of lower rural incomes). Urban physical environments may affect levels of drug abuse and drug deaths [36,37], e.g., by facilitating access to drugs. With regard to the US, Hedegaard et al. [38] report that “the age-adjusted rate of drug overdose deaths was higher in urban than in rural counties (22.0 and 20.0 per 100,000, respectively)”.

Supply side factors may also influence drug death levels, with higher mortality associated with easier access. For example, Monnat [39] investigates opioid supply factors (exposure to prescription opioids and fentanyl supply) as influences on US county drug mortality over 2014–2016. In the US, increases in opioid supply, through over-prescribing or illegal access, have been cited as a factor explaining increased opioid-related mortality, as opposed to job losses and “deaths of despair”. Ruhm [40] investigates “the alternative hypothesis that changes in the drug [supply] environment are a main cause of rising overdose deaths”. He finds a significant impact of opioid prescribing patterns on drug deaths at US county level, after adjusting for incomplete reporting of drug involvement on death certificates. Relevant also to the influence of supply factors in the US is a demarcation of phases of the opioid crisis. Thus, DeWeerdt [41] argues that “The opioid epidemic has had three phases: the first was dominated by prescription opioids, the second by heroin, and the third by cheaper—but more potent—synthetic opioids such as fentanyl. All of these forms of opioid remain relevant to the current crisis”. Excess prescribing of opioids has also occurred in the UK [42] and Canada [43].

## 3. Data and Methods 

The US has had one of the world’s fastest growth rates in drug-related deaths generally and opioid-related deaths in particular. Drug deaths involving any form of opioid—prescription opioids, other synthetic opioids (such as fentanyl), and heroin—rose from 18,515 in 2007 to 47,600 in 2017, before declining slightly to 46,802 in 2018. The majority (around 70%) of deaths were among males. The age-standardized rate for all drug-related mortality rose from 6.1 per 100,000 in 1999 to 21.7 in 2017 [44].

We consider a regression relating drug-related deaths in US counties between 2015 and 2017 to area characteristics. Drug deaths were defined with ICD10 codes X40–X44, X60–X64, X85, and Y10–Y14 [44]. We apply a regression methodology appropriate to the form of the outcome, namely a positive count. This methodology is sometimes referred to as Bayesian disease mapping [45]. Specifically, we use a Poisson regression with log link, where the log link is needed to ensure that the regression produces a positive predicted response; this method is set out in Appendix A. We also allow for the fact that small death counts are not provided for some areas (see Appendix B). The goal of disease mapping is to estimate relative risks of drug-related death for each county in the US, where the US-wide relative risk is 1 [46]. For example, an extremely high relative risk might be 5, and a low relative risk might be 0.1 or 0.2. The model involves known variables which are postulated risk factors but also includes two sets of random effects: one set represents spatially correlated but unobserved risk factors, the other represents Poisson extra-heterogeneity (also called over-dispersion) since the variance of drug deaths exceeds the mean number of drug deaths. 

We also study whether regression coefficients vary over the USA. If there were such variability, then this would be an example of geographic heterogeneity. Classical methods to investigate this include geographically weighted regression [47,48], whereas the Bayesian approach, adopted here, involves spatially varying regression through use of random effects [49,50].

Spatial correlation in relative risks of drug deaths is to be expected [51,52], and we can assess this from the model. Summary measures such as Moran’s I can be used. We can also apply a Bayesian estimator of local indicators of spatial association (LISA) clustering [53], used by Wilt et al. [54] in connection with drug deaths. We obtain the probability that county i is a high-high cluster core, namely a high mortality county surrounded by similarly high mortality counties. These probabilities can also be obtained for low-low clusters, where a low mortality county is surrounded by similarly low mortality counties.

There are seven variables, X_1_ to X_7_, postulated as risk factors for drug deaths. We consider two alternative specifications for X_1_, the variable used to measure employment opportunities. The first specification uses the 2016 county unemployment rate, while the second uses the percent point difference between unemployment in 2016 and in 2006. 

X_2_ serves both as an indicator of area socio-economic status and a measure of within county income disparities. It is an index of concentration at extremes, abbreviated as ICE [55], obtained from the 2016 American Community Survey as [(t.high)_i_ − (t.low)_i_]/T_i_, where T_i_ is total households in county i, (t.high)_i_ is the number of households with incomes over $150,000 and (t.low)_i_ is the number of households with incomes below $15,000. The highest and lowest scores on this index are for Loudoun (Virginia) and Holmes county (Mississippi), respectively. This variable was found to be a clearer measure of area socio-economic status in the regression than county poverty rates. 

X_3_ is a measure of social capital based on the index of Rupasingha et al. [31]. X_4_ is a measure of urban-rural status, namely the 2010 Census proportion of county population living in rural areas. X_5_ is a measure of racial-ethnic segregation from the County Health Rankings (https://www.countyhealthrankings.org/); this is an index of dissimilarity where higher values indicate greater residential segregation between non-white and white county residents.

X_6_ and X_7_ are measures of drug availability and supply and are at state level. X_6_ is an indicator of illicit supplies of fentanyl [56] calculated using data collected under the National Forensic Laboratory Information System (NFLIS) scheme. This is calculated as a ratio of seizures to the population aged 15–64; this age group is used to correct for the impact of population size as illegal opioid use is concentrated in these ages and tails off sharply among the over 65 s [57]. X_7_ is a measure of legal opioid prescribing [58], though it should be noted that legal prescription opioid use is a risk factor for illicit drug use—for example, of heroin [59]. As noted in [58], prescribing rates for opioids vary widely, and while the overall opioid prescribing rate in 2018 was 51.4 prescriptions per 100 people, some areas have rates six times higher than that.

We use the program WINBUGS [60] (MRC Biostatistics Unit, University of Cambridge, Cambridge, UK) to carry out estimation of the model. Estimation involves two chains to 20,000 iterations, with assessment of convergence based on Brooks–Gelman–Rubin diagnostics [61]. Comparisons of fit between models are based on the WAIC (Watanabe–Akaike information criterion) [62], which is lower for better fitting models. This is relevant to comparing the alternative specifications for job availability, namely a model based on unemployment in 2016 and a model based on percent point differences in unemployment between 2006 and 2016.

## 4. Results

### 4.1. Regression Findings

Table 1 shows the Poisson regression coefficients of the postulated risk factors under the alternative models for employment opportunities. The respective WAIC values on the unemployment and unemployment growth models are 20,315 and 20,356, so the unemployment model (top panel in Table 1) performs best. 

The risk factors listed in Table 1 are in a [0,1] form, with the value 1 corresponding to the maximum score, and 0 to the minimum score. Since the risk factors are on a common scale, the regression coefficients can be directly compared to assess their relative importance in explaining variation in risks of drug-related death between counties. The Poisson regression involves a log link model for relative risks of drug death. Thus, exponentiating the regression coefficients shows the relative risk of drug death when the county with the highest score on, say, social capital, is compared with the county having the lowest score. Thus, from Table 1, counties with high social capital have around half (0.57) of the risk of drug death than those with low social capital.

The highest coefficients (in absolute terms) are for social capital and area income disparities (high vs. low income groups). High levels of social capital in a county reduce the risk of drug-related death, and the same applies to counties with high percentages of households with incomes over $150,000 and/or low percentages of households with incomes under $15,000. Remaining risk factors all have significant effects (in the sense that their 95% intervals are confined either to negative or to positive values) but have lesser impacts than social capital and area income contrasts.

### 4.2. Spatial Clustering in Drug Death Risks

As discussed above, we can assess the extent of spatial clustering in risks of drug-related deaths. A simple summary of spatial clustering is provided by Moran’s I (e.g., [63]), obtained using the R program spdep and the moran.test option. We find a high Moran value for estimated relative risks of drug-related deaths, namely 0.72 (sd 0.01). This partly reflects high spatial correlation in the risk factors: social capital, unemployment, and ICE indicators have respective Moran values of 0.50 (0.01), 0.63 (0.01), and 0.61 (0.01). Lesser correlation shows for race segregation and rurality, respectively 0.29 (0.01) and 0.32 (0.01).

Regarding Bayesian estimation of LISA clusters, as part of the estimation process, we can determine which counties have an over 95% probability of being high risk (i.e., a relative risk over 1) for drug-related mortality. Similarly, we can determine which areas have a more than 95% probability of being high risk cluster centers; that is, not only is the county itself a high risk area but so also is the set of counties adjacent to it. This is a “high mortality area near high mortality” in the terminology of Wilt et al. [54]. Analogously, we can determine counties which have high probabilities of being low risk and of being low mortality near low mortality. 

### 4.3. Skew Patterns and Spatial Concentration in Drug Death Risk

Table 2 shows the locations of such counties according to US state. One thing to notice immediately is that whereas only 585 of 3141 counties (or 19%) are definitively high risk, 1873 (or 60%) are definitively low risk—that is, the geographic risk pattern is highly skewed. High risk of drug-related death is highly concentrated geographically in the United States, whereas low risk is the pattern typical of much of the USA. 

High risk counties near other high risk counties account for the majority (429 out of 585) of high risk counties. There are a relatively small number of counties (125) which are spatial outliers, namely high risk themselves but surrounded by counties which are not definitively high or low risk. There is also a small number (31) of high-low counties, where a high risk county is surrounded by low risk areas. 

**Figure 1:**
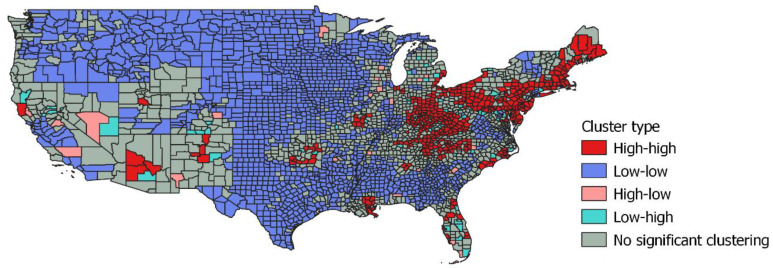
Continental USA. Drug-related mortality 2015–2017, cluster types.

Figure 1 shows that high risk clusters are mainly in the northeast, extending into some adjacent midwest states (Ohio) and to some southeast states (Kentucky, Tennessee, Delaware, Maryland, West Virginia) including Appalachia. The exceptions to high risk in the northeast are more rural counties in New York and Vermont. Other locations of localized high risk are in Arizona, New Mexico, and Oklahoma. 

**Figure 2:**
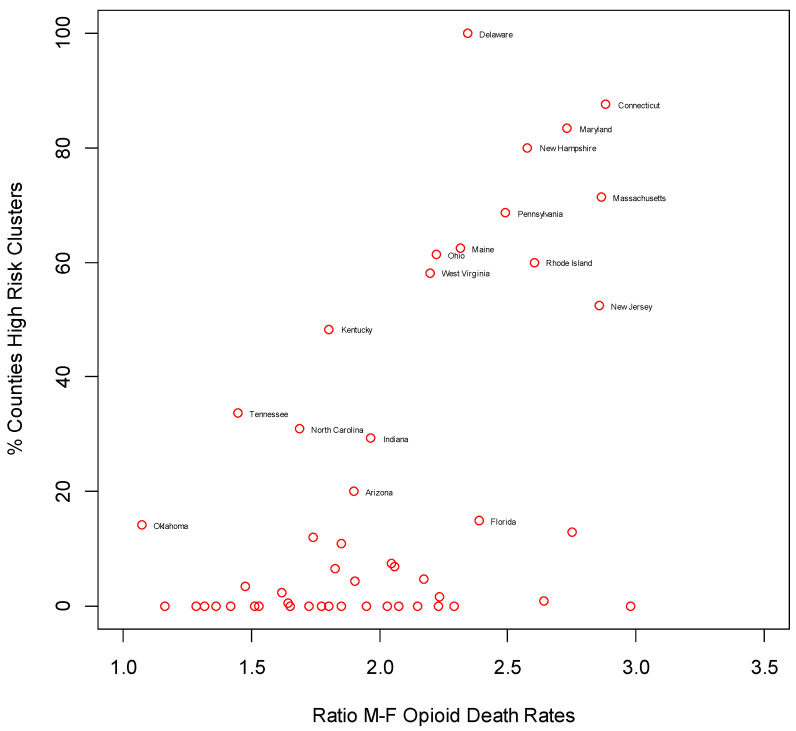
Male–female opioid mortality ratio and high risk drug death clustering.

There is evidence from other studies that rates of drug-related death are elevated among males [64]. Here, we find a correlation of 0.55 at state level between the ratio (in 2016) of male to female opioid age standardized death rates and the proportion of counties in each state classed as high risk cluster centers in Table 2. This relationship, shown graphically in Figure 2, suggests that analysis of drug deaths by gender would show high risk clustering as especially apparent for males.

### 4.4. Spatially Varying Coefficients

When geographic heterogeneity in covariate impacts is allowed for (in the better fitting unemployment model), we find a major gain in fit, with the WAIC reduced to 18,329. Table 3 summarizes the coefficient variation (for the five covariates which are observed at county level) in terms of averages for the nine US Census Divisions. 

It can be seen that the average effects of unemployment and segregation are attenuated as compared to Table 1, whereas average impacts of income disparity, social capital, and rurality are enhanced. Regarding contrasts between Divisions, one can see a different explanatory pattern in (say) the Pacific Division, where, compared to the average, social capital and rurality are more important, and income disparities and segregation less important, as influences on drug death rates.

## 5. Discussion

Analysis of small area contrasts in drug-related deaths has raised differing themes according to the country where research takes place. In the US, where there is extensive ecological research into drug-related deaths, different phases (with different causal influences) have been identified [41,65]. 

Thus, Zoorob [65] highlights the role of fentanyl in recent increases in drug-related mortality. Since fentanyl access is higher in the Eastern US, Zoorob argues that “the epicenter of the overdose crisis shifted towards the eastern United States over these years” as part of the third wave of the overdose epidemic. A similar trend, looking at changes in the clustering of high risk of drug death, is identified by Wilt et al. [51]. 

In terms of implications for intervention and funding, drug law enforcement [66], and drug abuse prevention, the locations of high risk clusters are important. The analysis above (e.g., see Figure 1) is consistent with findings by other studies in showing a geographic skew in high risk clustering towards the east and northeast of the US, while most of the rest of the US is low risk. A spatial concentration of drug-related deaths appears in studies of other countries [28].

The clustering analysis in our US study is conditional on a particular regression model. In this model, relative risks of drug death in different US counties are modeled as a function of income disparity within counties, job availability, social capital, levels of rurality, racial segregation, and measures of supply. None of these risk factors have a predominant influence. By contrast, many explanations are predominantly uni-causal. For example, the “deaths of despair” concept places a strong emphasis on the loss of traditional employment opportunities.

Regarding the research evidence on employment availability, a number of US studies find unemployment, or unemployment increases, to be associated with drug-related mortality or related outcomes [19,67], and there are reports of similar findings in other high-income societies such as Australia [68]. At the individual level, psychological distress is proposed as a mediator variable [69].

However, the analysis here, while finding unemployment, and increases in unemployment, to have a significant influence on raising drug-related deaths (at least before allowing for coefficient variation), does not show an overwhelming impact for these variables. Rather, factors such as social capital, income levels, race segregation, and drug supply are also important. An urban bias to US drug-related deaths is also confirmed. Thus, the evidence here is of a set of relevant area risk factors, as against a uni-causal explanation.

Support for a more nuanced multifactorial explanation of US trends is provided by recent overviews [29,70,71], stressing the impact of social as well as economic conditions, of community resilience, and of supply as well as demand factors. Thus, quoting [29], “strong communities offer resilience against drug epidemics”.

The same relevance of social environment is true of other high-income countries where ecological studies of drug death have been carried out. A study of drug deaths in Scotland [28], where drug-related deaths have risen as fast as they have in the US, showed that the impact of deprivation on drug-related deaths is moderated by social cohesion, which is measured inversely by an index of social fragmentation. Work on opioid use in Australia stresses the role of both demand and supply factors [72] and community resilience [73]. 

The analysis in this paper has had to allow for a relatively high rate of missing data (non-release of small death counts, namely counts under 10). The counties affected are mainly rural counties with small populations. One strategy [74] is to omit such counties from the analysis, but such a “complete cases” analysis may lead to bias [75], and may in particular affect inferences about area characteristics which affect missing data rates. Thus, [74] report higher drug deaths in rural counties, whereas the present analysis, which explicitly models the missing data mechanism, finds rural areas to have lower drug mortality.

## 6. Conclusions

Pronounced intra-national variations in drug-related mortality parallel international differences; see, for example, the studies [32,76] on Canada and Italy, respectively. There are unique national aspects to explaining contextual variations. For example, fentanyl supply is most important in the US and Canada [76], whereas in Germany, supplies of methamphetamine are important [77]. However, common themes are also apparent, such as community resilience offsetting impacts of job loss, area socioeconomic status, or area poverty. 

The present study on US small area variations supports a multifactorial explanation, with area income levels (and income disparity within areas) and social capital as paramount influences. Rurality is also a significant influence and has a negative impact when other area risk factors are controlled for (i.e., when correlations between area predictors are controlled in regression). The analysis here downplays the impacts of unemployment or unemployment change, especially when geographic heterogeneity in predictor impacts is allowed for in a spatially varying coefficients model. We also find a considerable geographic skew in the location of high risk clustering in the US [78].

The analysis is subject to certain limitations. The first, and one that must always be acknowledged with regard to ecological (area) studies, is that causal influences regarding individual health risk factors cannot be inferred from ecological studies. On the other hand, there is strong evidence that place (or contextual) effects per se, and interactions between individuals and environments, are relevant to explaining geographic variations in drug deaths [9,79]. 

A further caveat to making international comparisons in contextual effects is that the international evidence base on contextual variation in drug-related mortality is relatively limited. There are considerable research gaps in the small area study of drug-related deaths, with the great majority of studies being for the US. 

Other caveats with regard to the US analysis concern especially the available data. First, and as noted by [80], “the methods used to classify deaths on death certificates may be leading to a substantial undercount of these deaths [in the US]”, and later, “about 25% of U.S. overdose deaths had no drugs specified on the death certificate, so it is likely that national statistics underestimate by a substantial fraction the number of opioid analgesic- and heroin-related deaths”. The second problem with the data is the non-release of small death counts (counts under 10), so conclusions obtained may be affected by the strategy used to deal with this.

## Figures and Tables

**Table 1:** Covariate effects. Coefficients and relative risks.

Model Version and Area Predictor	Estimated Coefficient	2.5%	97.5%	Relative Risk of Drug-Related Mortality (Highest vs. Lowest County Score)	2.5%	97.5%
*Unemployment Model*						
Constant	−0.12	−0.23	−0.03			
Unemployment	0.33	0.06	0.59	1.39	1.07	1.81
ICE (High vs. Low Incomes)	−0.58	−0.70	−0.42	0.56	0.50	0.66
Social Capital	−0.57	−0.96	−0.21	0.57	0.38	0.81
Rurality	−0.31	−0.40	−0.23	0.73	0.67	0.79
Race Segregation	0.36	0.26	0.45	1.43	1.29	1.57
Opioid Prescribing	0.17	0.05	0.26	1.18	1.05	1.81
Fentanyl Seizure Rate	0.19	0.04	0.37	1.21	1.04	1.30
*Growth in Unemployment Model*						
Constant	−0.14	−0.28	−0.01			
Growth in Unemployment	0.25	0.07	0.41	1.29	1.08	1.51
ICE (High vs. Low Incomes)	−0.71	−0.84	−0.59	0.49	0.43	0.55
Social Capital	−0.51	−0.82	−0.14	0.60	0.44	0.87
Rurality	−0.29	−0.36	−0.21	0.75	0.70	0.81
Race Segregation	0.35	0.23	0.48	1.41	1.27	1.62
Opioid Prescribing	0.16	0.06	0.25	1.18	1.07	1.28
Fentanyl Seizure Rate	0.17	0.03	0.31	1.19	1.04	1.37

ICE: index of concentration at extremes.

**Table 2:** Distribution of Risk and Risk Cluster Status across US States.

Percent of Counties Which Are				
State	High Risk	Low Risk	High-High Cluster Centers	Low-Low Cluster Centers
Alabama	7	73	4	66
Alaska	0	86	0	82
Arizona	47	13	20	0
Arkansas	3	68	0	61
California	12	60	2	38
Colorado	6	78	0	55
Connecticut	88	12	88	0
Delaware	100	0	100	0
District of Columbia	100	0	0	0
Florida	27	34	15	18
Georgia	6	72	1	67
Hawaii	0	100	0	100
Idaho	0	82	0	77
Illinois	8	57	1	47
Indiana	37	29	29	16
Iowa	0	95	0	95
Kansas	0	94	0	94
Kentucky	52	24	48	12
Louisiana	14	56	11	52
Maine	56	0	63	0
Maryland	88	12	83	4
Massachusetts	71	7	71	0
Michigan	22	35	5	17
Minnesota	1	87	0	87
Mississippi	4	84	2	79
Missouri	10	63	7	55
Montana	0	96	0	96
Nebraska	0	100	0	100
Nevada	24	59	0	12
New Hampshire	80	0	80	0
New Jersey	57	24	52	19
New Mexico	30	33	12	6
New York	24	37	13	27
North Carolina	38	23	31	6
North Dakota	0	100	0	100
Ohio	65	6	61	0
Oklahoma	26	43	14	23
Oregon	0	78	0	69
Pennsylvania	69	9	69	0
Rhode Island	60	0	60	0
South Carolina	13	43	7	41
South Dakota	0	100	0	100
Tennessee	45	20	34	5
Texas	0	94	0	93
Utah	21	45	3	17
Vermont	7	21	0	0
Virginia	18	57	8	41
Washington	5	64	0	56
West Virginia	62	27	58	4
Wisconsin	8	74	0	65
Wyoming	0	74	0	70
USA	19	60	14	51

**Table 3:** Varying Predictor Effects according to US Census Divisions (Posterior Mean Effects and 95% Intervals).

		Mean	2.5%	97.5%			Mean	2.5%	97.5%
Unemployment	Divn 1	0.13	−0.05	0.33	Income Disparity (ICE Index)	Divn 1	−0.79	−1.05	−0.48
	Divn 2	0.09	−0.07	0.27		Divn 2	−0.78	−0.96	−0.54
	Divn 3	0.09	−0.02	0.26		Divn 3	−0.78	−0.93	−0.62
	Divn 4	0.11	0.01	0.28		Divn 4	−0.80	−0.99	−0.66
	Divn 5	0.05	−0.08	0.22		Divn 5	−0.74	−0.89	−0.57
	Divn 6	0.06	−0.05	0.23		Divn 6	−0.75	−0.89	−0.59
	Divn 7	0.11	−0.01	0.28		Divn 7	−0.77	−0.94	−0.61
	Divn 8	0.14	0.02	0.31		Divn 8	−0.82	−1.02	−0.67
	Divn 9	−0.09	−0.32	0.14		Divn 9	−0.51	−0.74	−0.34
	US average	0.08	−0.01	0.24		US average	−0.75	−0.93	−0.59
Social Capital	Divn 1	−0.54	−1.10	0.04	Rurality	Divn 1	−0.41	−0.52	−0.27
	Divn 2	−0.53	−1.10	−0.01		Divn 2	−0.41	−0.51	−0.32
	Divn 3	−0.50	−1.05	−0.03		Divn 3	−0.43	−0.50	−0.34
	Divn 4	−0.50	−1.04	−0.07		Divn 4	−0.44	−0.53	−0.33
	Divn 5	−0.51	−1.07	−0.04		Divn 5	−0.40	−0.45	−0.34
	Divn 6	−0.51	−1.06	−0.05		Divn 6	−0.39	−0.45	−0.33
	Divn 7	−0.49	−1.02	−0.05		Divn 7	−0.39	−0.48	−0.31
	Divn 8	−0.47	−1.02	−0.03		Divn 8	−0.44	−0.55	−0.35
	Divn 9	−0.73	−1.45	−0.15		Divn 9	−0.55	−0.72	−0.39
	US average	−0.53	−1.10	−0.06		US average	−0.43	−0.50	−0.36
Segregation	Divn 1	0.22	0.00	0.41	Definitions				
	Divn 2	0.21	0.04	0.36	Division 1	New England			
	Divn 3	0.20	0.08	0.32	Division 2	Mid-Atlantic (New Jersey, New York, Pennsylvania)			
	Divn 4	0.17	0.05	0.29	Division 3	East North Central (Illinois, Indiana, Michigan, Ohio, Wisc)			
	Divn 5	0.19	0.03	0.33	Division 4	West North Central			
	Divn 6	0.18	0.05	0.33	Division 5	South Atlantic			
	Divn 7	0.14	0.02	0.29	Division 6	East South Central (Alab., Kentucky, Miss., Tennessee)			
	Divn 8	0.16	−0.02	0.31	Division 7	West South Central (Ark., Louisiana, Okl., Texas)			
	Divn 9	0.10	−0.20	0.37	Division 8	Mountain			
	US average	0.17	0.03	0.31	Division 9	Pacific (Alaska, California, Hawaii, Oregon, Washington)			

## Appendix A. Regression Methods

We adopt Bayesian disease mapping, namely a form of generalized linear model to estimate relative risks of drug-related mortality in different areas. The response is the number of deaths in each county over a 3-year period (2015–2017), and a Poisson regression is adopted, with a log link model incorporating risk factors *X_i_* for county *i* (e.g., unemployment rates) [78]. For some counties, the death data are missing (see Appendix B), necessitating specialized techniques. The regression includes random effects to reflect spatially structured but unobserved risk factors, and also a separate set of random effects to take account of Poisson over-dispersion—since the variance of the death counts (50,987) exceeds the average count (105).

Thus, for counts Y_i_ of drug-related deaths for counties *i* = 1, …, *N*

*Y_i_* ~ Poisson(*E_i_ρ_i_*) (A1)

log(*ρ_i_*) = *X_i_β* + *s_i_* + *u_i_* (A2)

where *E_i_* are expected drug deaths, *ρ_i_* is the relative risk of drug death for county *i*, *β* are regression coefficients for risk factors, *s_i_* are random effects representing unobserved but spatially correlated risk factors, and *u_i_* are iid random effects. Expected deaths are known offsets obtained by multiplying county populations in 2016 by US-wide age-specific drug death rates. The random effects *s_i_* are assumed to follow the scheme of Besag et al. [81], in which the spatial effect for area *i* depends on spatial effects in adjacent areas. The predictors *X* are all converted to a [0,1] scale, so that their relative importance as risk factors can be assessed.

In the spatially varying coefficients model, the regression effect for predictor p and county *i* is specified as *b_pi_* = *β_p_* + *s_pi_*, where the *s_pi_* are zero mean spatial effects as in [81], and *β_p_* is a fixed effect. This is implemented in WINBUGS using the car.normal function for spatially correlated effects.

The data are overdispersed relative to the Poisson, with mean death count exceeding the variance. One option is a negative binomial model instead. However, the negative binomial is one of a broader class that can be obtained by introducing multiplicative random effects into a Poisson model [82,83]. Thus, one has that the Poisson means for incidence are specified by

*μ_i_* = *E_i_ρ_i_ λ_i_*

where *λ_i_* are positive random effects. For the Poisson-gamma model (equivalent to a negative binomial), the *λ_i_* are gamma distributed with mean 1. Another option is to take log(*λ_i_*) as normally distributed, leading to a Poisson-lognormal model for overdispersion. Specifically, under the log-link in (A1)–(A2), we have that *u_i_* = log(*λ_i_*) is normal, giving a Poisson-lognormal.

## Appendix B. Missing Death Data

In counties with death counts Y_i_ not supplied (the CDC Wonder site does not release death totals under 10), the sampling of the missing totals Y_i_ is subject to an upper threshold of 9. Expected counts are known for these counties, and sampling is from the relative risk model (A1), so taking account of known covariates for these counties, and also borrowing strength from neighboring counties, thereby accounting for spatial correlation in unknown factors affecting relative risks. A model for informative (or non-ignorable) missing data is also adopted [84], since the chance of death data being missing is clearly related to the size of the death count that would have been observed in the absence of thresholding. The probability of a count being missing is related to two covariates: *Z*_1_, a binary indicator, from (A1), of whether *ρ_i_* exceeds 1; and *Z*_2_, the proportion rural in the county, since rural counties tend to have both low relative risks and low populations.

We carry out a logit regression with response *M_i_* = 1 for missing mortality counts, and *M_i_* = 0 otherwise. We expect the chance of a missing response to be negatively related to the binary indicator of high relative risk, and positively related to the county percent rural. Note that this is a selection model [84], with the chance of missing data conditional on the modeled mortality outcome. Thus, for the best fitting model under (A1), with unemployment rates to represent employment opportunities, we find posterior means (sd) for coefficients on *Z*_1_ and *Z*_2_ to be −4.15 (0.26) and 4.83 (0.22), respectively.

One way of gauging the success of the modeling strategy is to compare estimated relative risks at state level (from the model) with the age-adjusted death rates (over 2015–2017) from the CDC Wonder site. State death totals are not affected by any thresholding of death counts. The estimated state level relative risks are obtained by summing the Poisson county level model means *μ_i_* = *E_i_ρ_i_* and expected deaths *E_i_* within states *j* = 1, …, 51 and comparing the resulting two summed totals. We find a correlation of 0.996 between the 51 age-adjusted death rates and the estimated state relative risks of drug death (see Appendix B Table A1). There is a correlation of 0.999 between the moment estimators *Y_j_*/*E_j_* of relative risk at state level and the modeled estimates *μ_j_*/*E_j_*.

**Table A1:** Age Adjusted Death Rates and Modelled Relative Risks for US States (2015–2017).

	Deaths	Age Adjusted Death Rate per 100,000	Total μ	Total Expected Deaths	Modelled Relative Risk
Alaska	397	17.7	387.0	437.8	0.88
Alabama	2327	16.6	2389.0	2814.6	0.85
Arkansas	1239	14.4	1288.2	1693.8	0.76
Arizona	4188	20.5	4185.0	3811.6	1.10
California	14,181	11.4	14,188.2	22,966.6	0.62
Colorado	2826	16.5	2796.7	3219.3	0.87
Connecticut	2843	26.8	2838.9	2127.2	1.33
Distr. Columbia	704	33.9	695.1	430.8	1.61
Delaware	818	29.9	822.5	543.8	1.51
Florida	13,044	21.7	13,032.9	11,544.2	1.13
Georgia	4233	13.6	4294.6	5958.2	0.72
Hawaii	563	12.6	560.1	827.8	0.68
Iowa	964	10.8	994.8	1760.4	0.57
Idaho	697	14.6	681.4	913.9	0.75
Illinois	7024	18.2	7043.5	7595.5	0.93
Indiana	4623	24.3	4661.5	3801.5	1.23
Kansas	975	11.6	974.3	1642.4	0.59
Kentucky	4258	33.6	4223.5	2580.1	1.64
Louisiana	2965	21.8	2943.1	2708.6	1.09
Massachusetts	6119	30.1	6117.3	4047.6	1.51
Maryland	5576	30.1	5555.0	3574.3	1.55
Maine	1046	28.1	1074.9	786.8	1.37
Michigan	7021	24.2	7089.5	5754.9	1.23
Minnesota	1986	12.1	1993.3	3189.8	0.62
Missouri	3804	21.6	3828.9	3508.5	1.09
Mississippi	1057	12.2	1106.3	1709.6	0.65
Montana	376	12.4	369.1	586.4	0.63
North Carolina	5937	19.9	5977.8	5829.5	1.03
North Dakota	206	9.5	187.0	421.1	0.44
Nebraska	398	7.2	406.2	1066.4	0.38
New Hampshire	1370	36.8	1372.1	796.9	1.72
New Jersey	6195	23.2	6195.3	5310.5	1.17
New Mexico	1494	25.1	1456.4	1183.6	1.23
Nevada	1960	21.2	1930.5	1686.5	1.14
New York	10,313	17	10,370.9	11,799.5	0.88
Ohio	12,750	38.5	12,811.0	6730.3	1.90
Oklahoma	2313	20.2	2265.4	2212.0	1.02
Oregon	1541	12.1	1570.6	2352.8	0.67
Pennsylvania	13,279	36.1	13,305.5	7479.3	1.78
Rhode Island	956	30	960.1	625.6	1.53
South Carolina	2648	18.1	2698.7	2805.4	0.96
South Dakota	207	8.4	194.4	477.8	0.41
Tennessee	4863	24.5	4878.3	3836.3	1.27
Texas	8408	10	8459.9	15,645.0	0.54
Utah	1931	22.6	1910.3	1611.7	1.19
Virginia	3951	15.7	3999.4	4969.9	0.80
Vermont	358	20.7	371.3	369.2	1.01
Washington	3365	14.8	3354.9	4215.7	0.80
Wisconsin	3129	18.6	3131.8	3351.2	0.93
West Virginia	2583	50.3	2525.5	1075.8	2.35
Wyoming	264	15.4	258.7	338.8	0.76
USA	186,273	19.3	186,726.7	186,726.7	1.0

The WINBUGS code for the model is

model { for (*i* in 1:N) {Y[*i*] ~ dpois(mu[*i*]) I(,U[*i*])

mu[*i*] < −E[*i*] * rho[*i*]

# Indicator for high relative risk

high.rho[*i*] < −step(rho[*i*]−1)

# Clustering indicators (posterior means are estimates of probabilities)

hihi[*i*] < −step(rho[*i*]−1) * step(Locality.rho[*i*]−1)

lolo[*i*] < −step(1−rho[*i*]) * step(1−Locality.rho[*i*])

hilo[i] < −step(rho[*i*]−1) * step(1−Locality.rho[*i*])

lohi[i] < −step(1−rho[*i*]) * step(Locality.rho[*i*]−1)

# Locality average relative risk. cum.num are cumulative locality sizes

Locality.rho[*i*] < −sum(rho.adj[cum.num[*i*] + 1:cumnum[*i* + 1]])/num[*i*])

# Relative Risk Model

log(rho[*i*]) < −alpha + inprod(beta[], X[*i*]) + s[*i*] + u[*i*]

# unstructured (iid) random errors

u[*i*] ~ dnorm(0,tau.u)

# Missing Data Model

M[*i*] ~ dbern(pi[*i*])

Z[*i*,1] < −high.rho[*i*]

logit(pi[*i*]) < −alpha.M + inprod(gamma[], Z[*i*])}

# % of residual variation spatially structured

shspat < −100 * (sd(s[]) * sd(s[]))/(sd(s[]) * sd(s[]) + sd(u[]) * sd(u[]))

# Model for spatial random errors s[*i*]

# num[*i*] are numbers of neighbours of county *i*, adj is adjacency list

# NN is sum of num[*i*].

for (*i* in 1:NN) {weights[*i*] < −1; rho.adj[*i*] < −rho[adj[*i*]]}

s [1:N] ~ car.normal(adj[], weights[], num[], tau.s)

# Priors on Hyperparameters

tau.u ~ dgamma(1,0.01); tau.s ~ dgamma(1,0.01)

alpha ~ dnorm(0,0.01); alpha.M ~ dnorm(0,0.01)

for (*j* in 1:2) {gamma[*j*] ~ dnorm(0,0.1)}

for (*j* in 1:7) {beta[*j*] ~ dnorm(0,0.1)}}

The vector Y[] has NA for counties with missing responses; the vector U is set to 9 for these counties and to Yi when the death count is above the threshold of 9. The vectors adj and num can be derived in R by first inputting a shapefile using the maptools command, shape = readShapePoly((“shape.shp”), then using commands poly = poly2nb(shape) and WB = nb2WB(poly) to create lists WB$adj and WB$num. The vector cum.num can be obtained in R as

cum.num = c(); cum.num[1] = 0

cums=cumsum(WB$num)

for (*j* in 1:N) {cum.num[*j* + 1] = cums[*j*]}
